# Trauma Simulation Training Increases Confidence Levels in Prehospital Personnel Performing Life-Saving Interventions in Trauma Patients

**DOI:** 10.1155/2016/5437490

**Published:** 2016-08-03

**Authors:** Christine M. Van Dillen, Matthew R. Tice, Archita D. Patel, David A. Meurer, Joseph A. Tyndall, Marie Carmelle Elie, Jonathan J. Shuster

**Affiliations:** ^1^Department of Emergency Medicine, University of Florida College of Medicine, Gainesville, FL 32610, USA; ^2^Department of Health Outcomes and Policy, University of Florida College of Medicine, Gainesville, FL 32610, USA

## Abstract

*Introduction*. Limited evidence is available on simulation training of prehospital care providers, specifically the use of tourniquets and needle decompression. This study focused on whether the confidence level of prehospital personnel performing these skills improved through simulation training.* Methods*. Prehospital personnel from Alachua County Fire Rescue were enrolled in the study over a 2- to 3-week period based on their availability. Two scenarios were presented to them: a motorcycle crash resulting in a leg amputation requiring a tourniquet and an intoxicated patient with a stab wound, who experienced tension pneumothorax requiring needle decompression. Crews were asked to rate their confidence levels before and after exposure to the scenarios. Timing of the simulation interventions was compared with actual scene times to determine applicability of simulation in measuring the efficiency of prehospital personnel.* Results*. Results were collected from 129 participants. Pre- and postexposure scores increased by a mean of 1.15 (SD 1.32; 95% CI, 0.88–1.42; *P* < 0.001). Comparison of actual scene times with simulated scene times yielded a 1.39-fold difference (95% CI, 1.25–1.55) for Scenario 1 and 1.59 times longer for Scenario 2 (95% CI, 1.43–1.77).* Conclusion*. Simulation training improved prehospital care providers' confidence level in performing two life-saving procedures.

## 1. Introduction

The advancement of patient care is the final goal of all training in medicine. Over the past several decades, significant innovations have been introduced in the educational methodology and tools used to deliver clinical education. With the rapid advancement in technology in medical education, simulation has been shown to facilitate this type of learning, especially with regard to skills needed in the prehospital environment [1]. Simulation provides an optimal environment for learning and can refresh personnel on infrequently performed procedures and patient scenarios [1, 2]. It also allows high-risk scenarios to be repeated for multiple providers to focus on specific management skills and techniques as well as assessment by supervisors and medical directors in critical patient scenarios [1, 3]. In the prehospital setting, personnel in the field operate from medical care protocols. A medical director is often limited to chart review to assess performance of critical thinking, patient interaction, procedural performance, and overall quality of care provided. This method of review is extremely limited by self-reporting and documentation. As a result of these limitations, simulation has become a very useful learning and assessment tool for emergency medical services (EMS) agencies.

Recent meta-analyses focusing on medical simulation in general concluded that the presence of medical simulation in the medical education strategy greatly enhanced skills and knowledge compared with curricula that excluded simulation [4, 5]. Evidence focusing on simulated scenarios for prehospital personnel is still somewhat limited [1]. The literature involving the use of simulation with prehospital care providers broadly covers topics that include intubation, ventilation, trauma care, cardiopulmonary resuscitation, and resuscitation [1, 6]. In trauma simulation articles, specific assessment skills, triage management, and procedural performance are the topics often discussed. Specific procedural skills such as tourniquet application and chest decompression have received little attention in the medical literature [1]. This study described in this paper evaluated the effect of introducing prehospital personnel to simulated critical trauma patients requiring the emergent use of tourniquets and needle decompression on their confidence levels in performing these procedures. The purpose of this study is to evaluate if the confidence of prehospital personnel can be improved through simulation training.

Previous studies in the prehospital setting have used visual analog scales (VASs) to assess confidence in procedures [7]. Assessment of the efficiency with which paramedics recognized the need for emergent life-saving interventions (specifically needle decompression for pneumothorax and tourniquet placement for amputation) and recognized patients meeting Florida trauma alert criteria was also documented. This study also compared simulated scenario times with actual trauma scene times with regard to the timing of patient evaluation, interventions, and transport, which are key features that EMS medical directors review as part of their assessment of the provision of care to critical trauma patients.

## 2. Methods

Alachua County Fire Rescue (ACFR) provides EMS transport and first responder services in the county and staffs 12 full-time advanced life support (ALS) ambulances that cover 969 square miles and serve a general population of 246,336. Paramedics at ACFR are mostly male with an average of 12 years of service in the agency (Table 1). ACFR responds to approximately 36,000 calls annually. From June 2013 to June 2014, trauma was the primary reason for 2,209 of these calls, and an additional 216 were considered trauma alerts after field assessment. Care provided by crews is guided by medical protocols written and approved by the medical director. Quality assurance and improvement are done through a combination of online medical control (done via the radio), chart review, and a small amount of physician field response. Local system trauma alert criteria at the time of the study can be found in Table 2.

In this retrospective observational study, paramedic and emergency medical technician (EMT-basic) teams were presented with two scenarios. Scenario 1 was a motorcycle crash that caused the amputation of driver's right leg, requiring the life-saving intervention of placing a tourniquet on the limb. Scenario 2 involved an intoxicated patient who had sustained an occult stab wound in a bar fight and who later developed a tension pneumothorax requiring needle decompression. The crew members' perceived confidence levels in the treatment of these critically injured patients were evaluated via a self-assessment standard survey form before and after the presentation of the simulated scenarios. After each scenario, crew members received a debriefing about their performance and were trained to review both tourniquet placement and needle decompression as well as the associated indications, contraindications, and important treatment points. In previous studies, students who had experienced a posttraining debriefing felt that weaknesses and strengths discussed by observers enhanced their knowledge and clinical performance [6]. In our debriefing, clinical weaknesses and strengths as well as on-scene time utilization were discussed.

EMT-basic and paramedic crews came to the training facility during their shifts over a 2- to 3-week period based on the availability of the training teams. A total of 129 prehospital personnel participated in the training. The simulation sessions were presented in place of ACFR's quarterly training along with surveys and evaluations that were completed to assess the quality of the training model. In actual response scenarios, a minimum of an engine crew (one paramedic and two EMTs-basic) as well as a rescue crew (one paramedic and one EMT-basic) would usually be dispatched to the scene of a critical traumatic injury when adequate information is given at the time of dispatch. Therefore, each training group consisted of two to five crew members, depending on their availability. EMT-basic and paramedics participated in the simulation together; however data was only collected on the paramedics as they would normally perform the procedures and make the clinical decisions. We excluded those who were not available for training or did not complete the survey.

**Figure 1:**
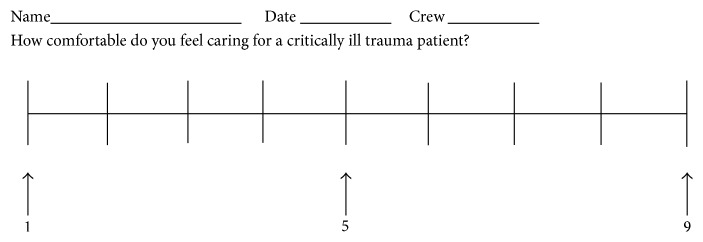
Visual analog scale (1–9).

Prior to the training, crews completed a survey that asked them to rate their confidence level when evaluating and treating critically injured patients, using a VAS of 1 to 9 (Figure 1). Identifying information was collected and used initially to correlate their pre- and posttraining survey results. Soon after the data were collected, identifying information was removed prior to analysis.

Training was initiated with crew members receiving a simulated call from dispatch for the initial case, along with a brief summary of the setting for their simulation. The simulator mannequin was then revealed to them so that they could start their assessment. Paramedic training officers from ACFR acted as evaluators to record the times of each intervention and to document whether critical actions were completed by crew members, the timing of these interventions, the timing of trauma alert notification, and the time spent on scene on a standard evaluation sheet. An emergency physician or emergency medicine resident was present to lead the scenario and answer questions regarding the patient's presentation. All physician and training captain team members received training on each scenario and evaluation sheet prior to the simulation to ensure that each group would be exposed to a standard scenario and evaluation process.

The simulation ended when the crew stated that they were leaving the scene to transport the patient or after 15 minutes had passed. The physician simulation leader and the evaluator then led a debriefing session with the crew members to discuss their performance. At this time, each crew received training on the clinical presentation of life-threatening hemorrhage in a trauma patient along with the indications and contraindications for placement of a tourniquet.

After completing the first scenario, the same crew was presented with the second scenario, undergoing active evaluation followed by a debriefing from the physician simulation leader and evaluator. This crew then received training on the identification of tension pneumothorax as well as the indications and contraindications for needle decompression. The postsimulation survey was then filled out by each individual crew member to indicate his or her postsimulation confidence level (a new copy of the same VAS was provided and completed by participants). They also recorded their subjective opinions about the simulation experience.

The primary outcome of interest was whether simulation scenarios increase confidence levels in prehospital care providers performing life-saving interventions on critically injured patients. A secondary outcome of interest was whether medical directors can use simulation as an accurate assessment tool to measure efficiency (the timing of interventions and the time spent on scene) of prehospital personnel at providing care for and transporting critically injured patients.

The evaluation sheet served as our data collection form during the simulation process. In addition, standard pre- and postsurvey sheets were provided to all crew members. These data were entered into Excel spreadsheets and deidentified manually. Analysis was performed using SAS 9.3 (Statistical Analysis Systems, Cary, NC). For timed variables, we used logs to estimate the typical fold changes once converted back to the original units by antilogs. This approach minimizes the adverse impact of outliers. All data collected were used to evaluate the training, not for personnel evaluations or corrective actions.

The data points collected are listed as follows:

- Did subjects enjoy the training?
- Did they want to participate in more simulation training?
- Total time spent on motorcycle crash scenario.
- Time on scene for motorcycle crash case.
- Total time spent on tension pneumothorax scenario.
- Time on scene for pneumothorax case.
- How comfortable do you feel when caring for a critically injured patient? (Confidence level before the scenario.)
- How comfortable do you feel when caring for a critically injured patient? (Confidence level after the scenario.)

## 3. Results

The providers' confidence levels were recorded in a survey via a VAS before and after participation in the training and then compared directly. When comparing each individual provider's scores, the score increased by a mean of 1.15 (SD = 1.32), with a 95% confidence interval of 1.15 ± 0.27, or from 0.88 to 1.42 for the mean change. This mean change is significant at *P* < 0.001 by a one-sample *t*-test on this paired difference. When asking participants whether they enjoyed the training, 99.0% reported positively and 98.0% reported that they would prefer more simulation training than the previous style of training.

To obtain a real-life comparison, scene times in trauma alert patients were calculated by looking at time of ambulance arrival on scene and ending with ambulance transport, using computer-assisted dispatch records over a 1-year period. When we compared true trauma alert scene times with our two simulated scenarios (Table 3), we found that actual scene times were significantly longer than times in the simulated scenarios. When comparing actual times with Scenario 1 simulated times, we found the point estimate and 95% confidence interval in the log scale is 0.3298 (0.2244, 0.4351). Upon taking antilogs, this converts to a 1.39-fold (1.25, 1.55) difference, with real scenarios being higher. In Scenario 2, we found the point estimate and 95% confidence interval in the log scale is 0.4646 (0.3593, 0.5699). Upon taking antilogs, this converts to 1.59-fold (1.43, 1.77) longer for the real scenario.

## 4. Discussion

Quality prehospital care is a critical function in the continuum of emergency care. In many scenarios, the prehospital phase of care is the health care system's earliest opportunity to improve patient outcomes during episodic care. The importance of creating effective models to develop expertise in prehospital care cannot be understated.

Prehospital personnel must be capable of providing life-saving interventions at a moment's notice. EMT-basic training is between 120 and 150 hours and EMT-paramedic between 1200 and 1800 hours in length, compared with the 2 to 4 years of training that nurses receive and the 11 to 13 years that physicians receive prior to being expected to evaluate and treat patients. Treatment decisions for time-sensitive emergency conditions must be made quickly and correctly; therefore, prehospital care providers must be well trained and have a high degree of confidence in initiating procedures under adverse circumstances. It can be inferred that higher provider confidence should lead to a decrease in time spent on scene because of the greater efficiency with procedures and decreased time to make a decision due to exposure to various scenarios from simulation. Our findings suggest that exposure to a critical trauma scenario requiring a life-saving intervention improves a prehospital care provider's confidence level in performing this procedure. Similar findings have been noted in other simulation studies, although not focusing specifically on tourniquet placement and needle decompression.

Overall, our results suggest that introducing simulation into EMS training can enhance the educational experience of prehospital care providers, allowing them to develop important experiential knowledge about procedures while gaining essential confidence in procedure performance. Previous evidence has suggested that education using clinical simulation can shorten the time it takes for trained personnel to become proficient in critical clinical procedures [6].

Our limitations include that the pre- and posttraining survey were a simple tool that was subjective in the assessment of confidence in performing procedures without specific evidence of outcomes or proficiency. In addition, the scene times documented in the simulation were much shorter than those in real trauma scenes in Alachua County. A clear limitation of the simulation environment is the inability to reliably create the chaos, distractions, and other stressors (e.g., interactions with other agencies and the public, extrication, and environmental factors) that are typically associated with real world situations. While this limitation is to some extent insurmountable and can clearly affect the ability of a simulation environment to precisely evaluate outcomes such as the true time spent on trauma calls, it does not preclude accurate measure of the prehospital care providers' assessments, decision-making, and action times, which directly impact actual scene times.

Future studies should focus on actual patient care outcomes as a result of such education interventions, which could be particularly helpful to rural EMS agencies and other municipalities with fewer scene calls.

In conclusion, this study demonstrates that the introduction of simulation into training can improve prehospital care providers' confidence in performing tourniquet placement and needle decompression in critically injured patients. These scenarios were chosen to test the prehospital personnel in their ability to make critical decisions and perform advanced procedures when necessary. Additional simulations could be done to assess other skills as well. These findings suggest that the integration of more simulation into prehospital care training may be useful, especially for rarely performed time critical life-saving skills.

## Figures and Tables

**Table 1:** Alachua county fire rescue paramedics' descriptive statistics.

Characteristics	Total # of paramedics (155)
Average age	40.1
Avg. years in service	12.4
Percent male	95%
Race	
White (not Hispanic)	93%
Hispanic/Latino (any race)	3%
Black/African American	4%

**Table 2:** 64J-2.004 adult trauma scorecard methodology.

*Adult trauma triage criteria and methodology*		
The EMT or paramedic will assess the condition of those injured persons with anatomical and physiological characteristics of a person sixteen (16) years of age or older for the presence of at least one of the following four (4) criteria to determine whether to transport as a trauma alert. These four criteria are to be applied in the order listed, and once any one criterion is met that identifies the patient as a trauma alert, no further assessment is required to determine the transport destination.		

*Criteria*		
(1) Meets color-coded triage system (see below)		
(2) GCS ≤ 12 (patient must be evaluated via GCS if not identified as a trauma alert after application of criterion 1)		
(3) Meets local criteria (specify)		
(4) Patient does not meet any of the trauma criteria listed above but, in the judgment of the EMT or paramedic, should be transported as a trauma alert (document)		

Component	Blue criteria (B)	Red criteria (R)

Airway^1^	± sustained RR ≥ 30	± active airway assistance^2^

Circulation	± sustained HR > 120	± lack of radial pulse with sustained fast heart rate (>120) or
		± BP <90

Best motor response	± BMR = 5	± BMR of ≤4 or
		± paralysis or
		± suspected spinal cord injury

Cutaneous	± tissue loss^3^ or	± amputation^4^ or
	± GSW to extremities	± 2^0^/3^0^ burns to >15% TBSA or
		± any penetrating injury to head, neck, or torso^5^

Longbone fracture	± single FX site due to MVA or	± multiple FX sites
	± fall > 10′	

Age	± ≥55	

Mechanism of injury	± ejection from vehicle or	
	± deformed steering wheel^6^	

(i) R: any *one (1)*-transport as a trauma alert

(ii) B: any *two (2)*-transport  as a trauma alert

^1^Airway evaluation is designed to reflect the intervention required for effective care. ^2^Not just oxygen. ^3^Degloving injuries, major flap avulsions (>5 in). ^4^Amputations proximal to the wrist or ankle. ^5^Excluding superficial wounds in which the depth of the wound can be easily determined. ^6^Only applies to driver of vehicle m: JR/PATTC/6/26/97.

**Table 3:** Scene time ranges for trauma alert patients.

*Scenario 1*	
Arrival on scene to trauma alert	0.25–9.96 min
Time to initiation of tourniquet placement	0.4–3.16 min
Total time on scene	5.33–9.96 min

*Scenario 2*	
Arrival on scene to trauma alert	1–8.03 min
Time to initiation of needle compression	2.2–5.76 min
Total time on scene	5.2–8.75 min
